# Supplementary material for: Caenorhabditis elegans Deficient in DOT-1.1 Exhibit Increases in H3K9me2 at Enhancer and Certain RNAi-Regulated Regions
Source: Cells. 2020 Aug 6;9(8):1846. doi: 10.3390/cells9081846 (PMC7464606; doi:10.3390/cells9081846)
Supplement: Supplementary file 1 [file cells-09-01846-s001.zip › cells-819834-supplementary for publication/cells-819834-suplementary figures-v4.pdf]

cells

MDPI

Supplemental Figures

Caenorhabditis elegans Deficient in DOT-1.1 Exhibit Increases in H3K9me2 at Enhancers and Certain RNAi-Regulated Regions

Ruben Esse [1,2] and Alla Grishok [1,*]

[1] Boston University School of Medicine, Department of Biochemistry, BU Genome Science Institute; Boston, MA 02118, USA; agrishok@bu.edu

[2] Present address: Department of Medical and Molecular Genetics, King's College London, London, SE1 9RT, United Kingdom

* Correspondence: agrishok@bu.edu

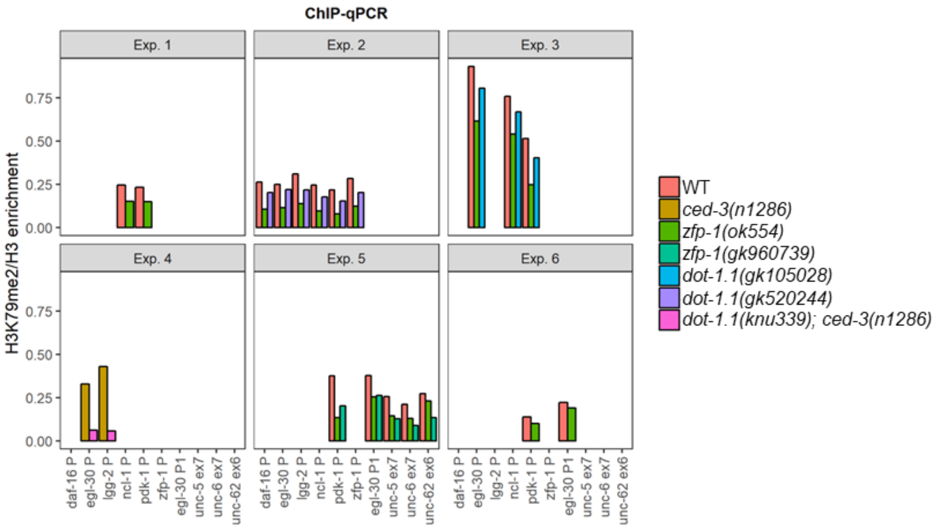

Figure S1. H3K79me2 ChIP-qPCR results showing decrease in H3K79me2 in various mutants used in this study. Six independent experiments are shown; the IP efficiency varies between the experiments, which is expected. Tested gene names are shown on the X axes, "P" after the gene name indicating the promoter region and "ex" indicating an exon region. The Y axis shows the ratio between the mean H3K79me2 ChIP-qPCR signal and the mean H3 ChIP-qPCR signal (from three technical replicates). The ChIP signals were computed as a "% of input". Note the background signal in *dot-1.1(knu339); ced-3(n1286)* in experiment #4, consistent with our published western blot data [1]. The H3K79me2 levels at the *pdk-1* promoter in *zfp-1(ok554)*, which is used for H3K9me2 ChIP-seq here, were assayed in five experiments shown and are ~50% reduced compared to wild type, consistent with our published work [2]. The results with *dot-1.1(gk520244)* and *zfp-1(gk960739)* alleles used in gonad migration experiments are shown in experiment #2 and experiment #5, respectively; note reduced H3K79me2 at the *unc-6* gene in *zfp-1(gk960739)* in experiment #5.

(a)

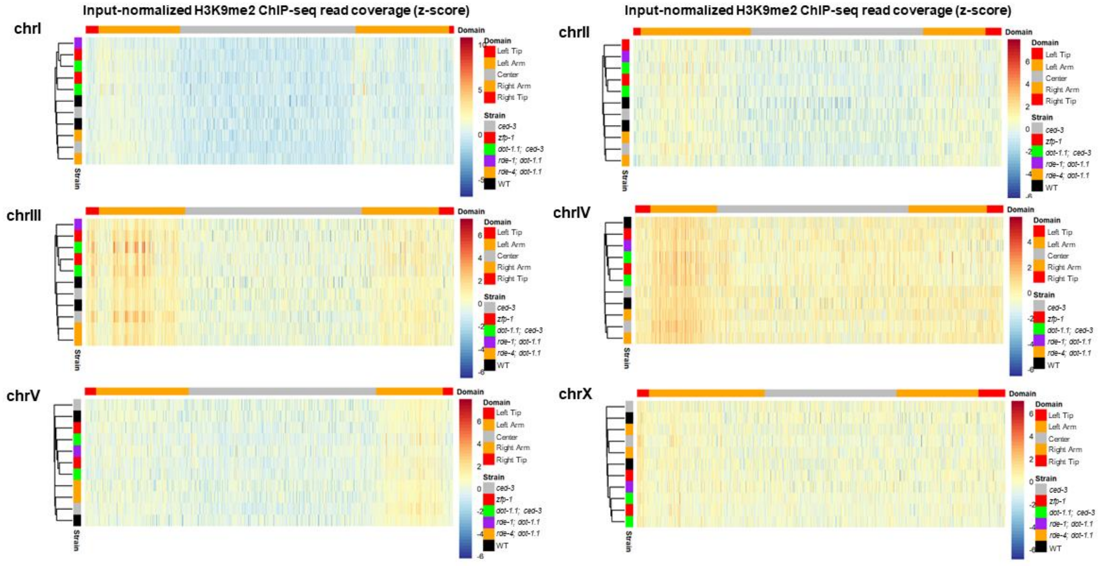

(b)

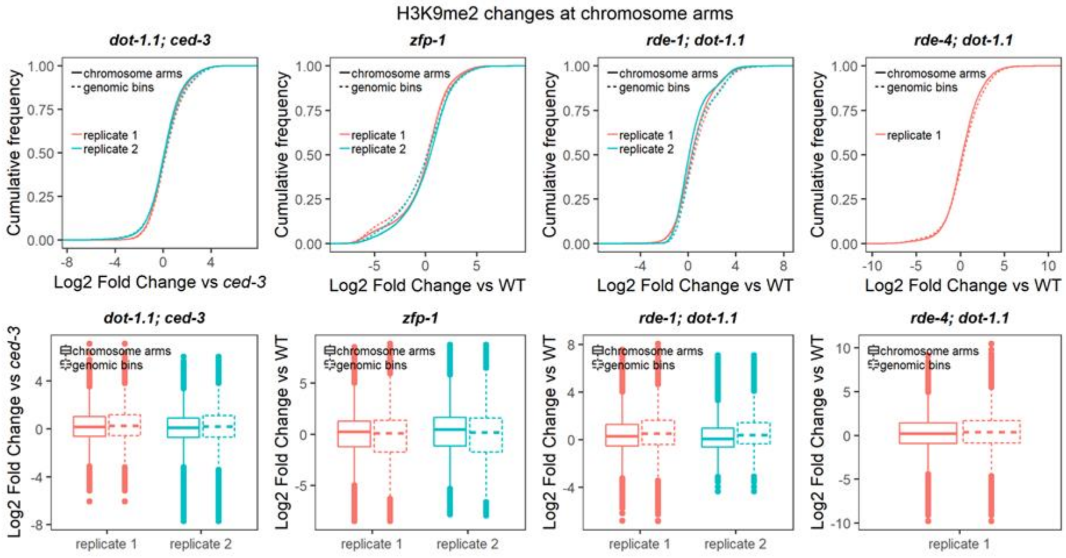

Figure S2. Deletion of DOT-1.1 does not disrupt preferential positioning of H3K9me2 at autosome arms. (**a**) Heatmaps showing coverage of H3K9me2 along the *C. elegans* chromosomes for each mutant analyzed. Input-normalized coverage (see Materials and Methods) was calculated for each of the 10,000 bp bins spanning each chromosome, and each bin was assigned a chromosome domain (chromosome domain coordinates are from [3]). (**b**) Cumulative distribution of the fold change (log2) of H3K9me2 ChIP-seq RPKM value (input-normalized) at the autosome arms between each mutant replicate and the average value in the corresponding background strain. Two-sample Kolmogorov-Smirnov (for cumulative distribution plots) and Wilcoxon rank sum (for boxplots) tests were performed to compare the cumulative changes between genomic bins located in chromosome arms and those in non-overlapping genomic bins spanning the genome. Statistical significance (p-values ranging between $< 2.2 \times 10^{-16}$ and 3.041×10^{-14}) was found for all the mutant replicates analyzed.

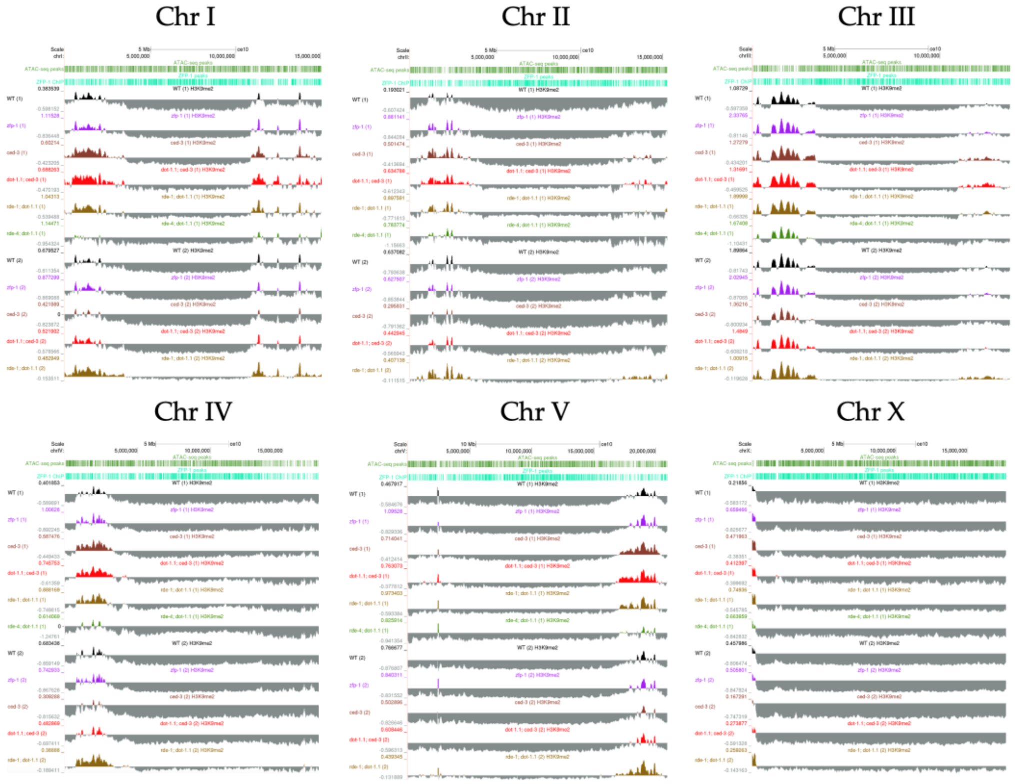

Figure S3. UCSC genome browser screenshots of H3K9me2 distribution along the six *C. elegans* chromosomes (not quantitative). ATAC-seq peaks: [4]. ZFP-1 peaks: modENCODE data, GEO submission GSE50301. H3K9me2 coverage tracks: our data.

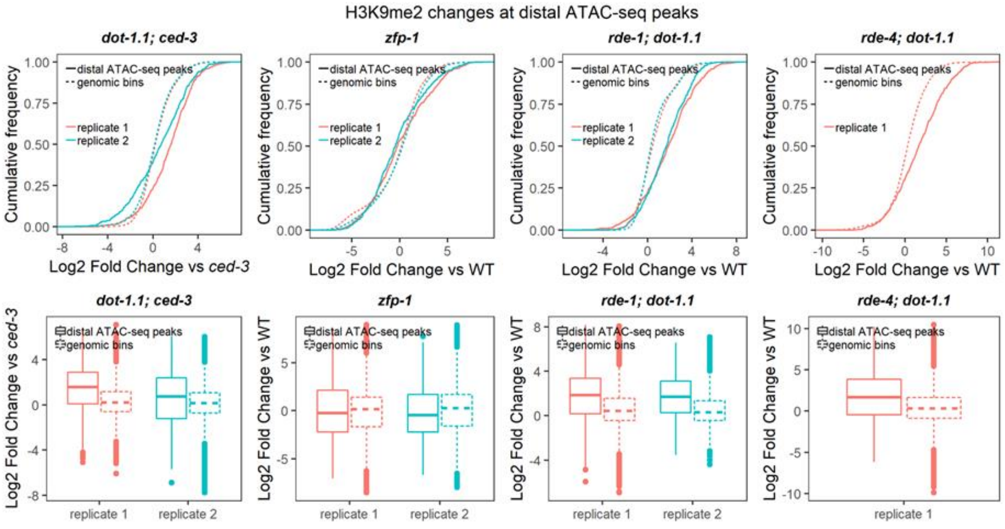

Figure S4. Global increase of H3K9me2 upon DOT-1.1 deletion at distal ATAC-seq peaks. Cumulative distribution of the fold change (log2) of H3K9me2 ChIP-seq RPKM value (input-normalized) at distal ATAC-seq peaks [4] between each mutant replicate and the average value in a background strain. Two-sample Kolmogorov-Smirnov (for cumulative distribution plots) and Wilcoxon rank sum (for boxplots) tests were performed to compare the cumulative changes between the distal ATAC-seq peaks and those in non-overlapping genomic bins spanning the genome. Statistical significance (*p*-values ranging between < 2.2×10^{-16} and 0.001017) was found for all *dot-1.1; ced-3*, *rde-1; dot-1.1* and *rde-4; dot-1.1* mutant replicates analyzed, as well as for the *zfp-1* mutant, with the exception of one of the replicates (Wilcoxon *p*-value = 0.2521).

(a)

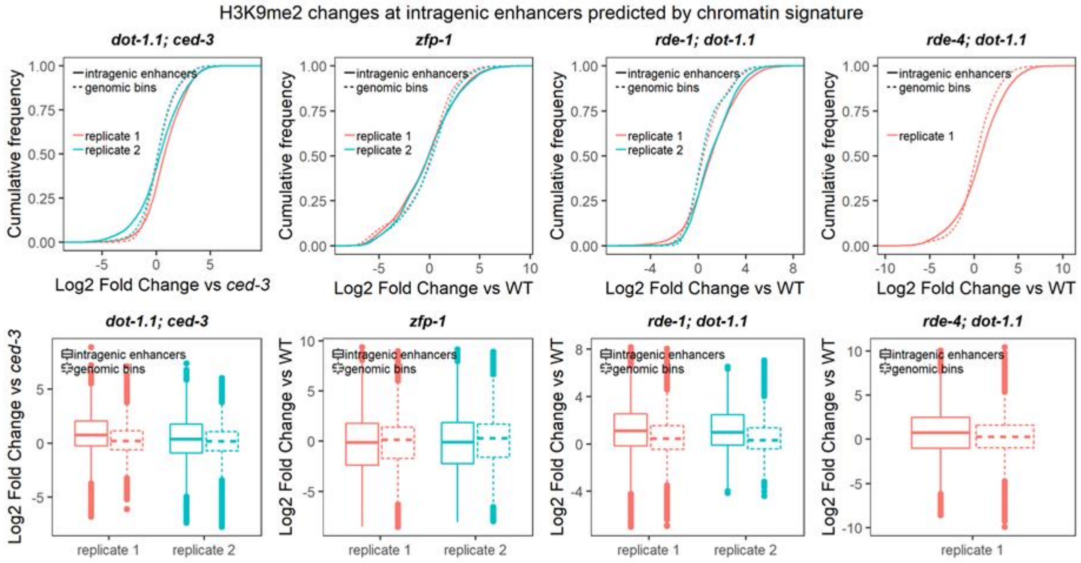

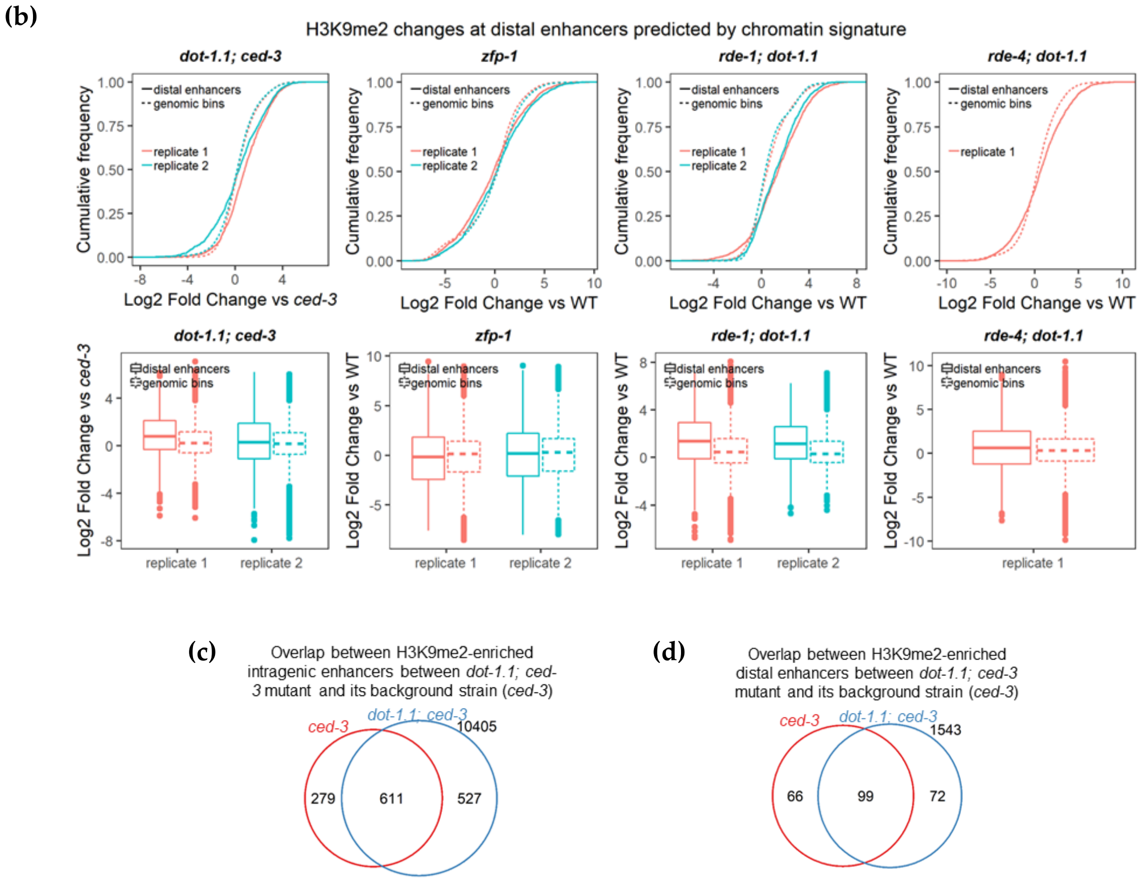

Figure S5. Deletion of DOT-1.1 leads to global increase in H3K9me2 at enhancers predicted by chromatin signatures. Shown are cumulative distribution plots and boxplots representing the fold change (log2) of H3K9me2 ChIP-seq RPKM value (input-normalized) at intragenic **(a)** and distal **(b)** enhancer domains [5] between each mutant replicate and the average value in the background strain. Two-sample Kolmogorov-Smirnov (for cumulative distribution plots) and Wilcoxon rank sum (for boxplots) tests were performed to compare the cumulative changes between enhancer domains and those in non-overlapping genomic bins spanning the genome. Statistical significance (p-values ranging between $< 2.2 \times 10^{-16}$ and 0.0072) was found for all *dot-1.1; ced-3*, *rde-1; dot-1.1* and *rde-4; dot-1.1* mutant replicates analyzed, as well as for the *zfp-1* mutant for intragenic enhancers, with the exception of one of the replicates (Wilcoxon p-value = 0.1447). The Venn diagrams show that the *dot-1.1; ced-3* mutant has more intragenic **(c)** and distal **(d)** enhancers overlapping with H3K9me2 peaks (observed in both replicates, see Materials and Methods) than the background strain (*ced-3* mutant). Numbers inside the circles designate enhancers overlapping with H3K9me2 peaks in either strain alone (left-most and right-most numbers) or in both strains (middle). Numbers outside the circles representing enhancers not overlapping with H3K9me2 peaks in any strain.

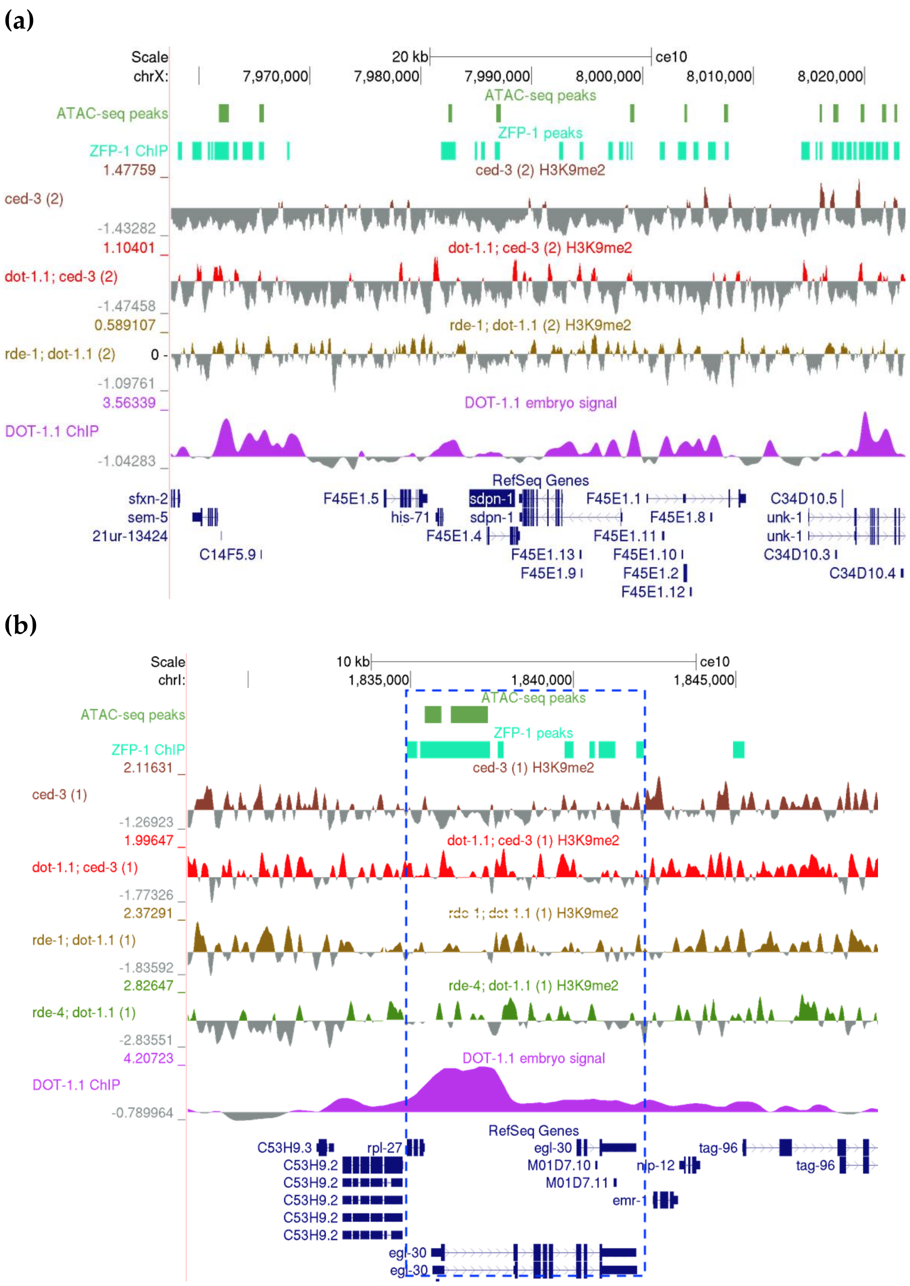

Figure S6. Examples of genomic loci containing developmental genes that gain H3K9me2 in the absence of DOT-1.1 (UCSC genome browser screenshots). (**a**) The ~60kb genomic region on ChrX containing many enhancers, including in the intron of the *sdpn-1* gene. (**b**) The *egl-30* locus bound by ZFP-1/DOT-1.1. ATAC-seq peaks: [4]. ZFP-1 peaks: modENCODE data, GEO submission GSE50301. H3K9me2 coverage tracks: our data. DOT-1.1 ChIP-chip signal: modENCODE data, GEO submission GSE37488.

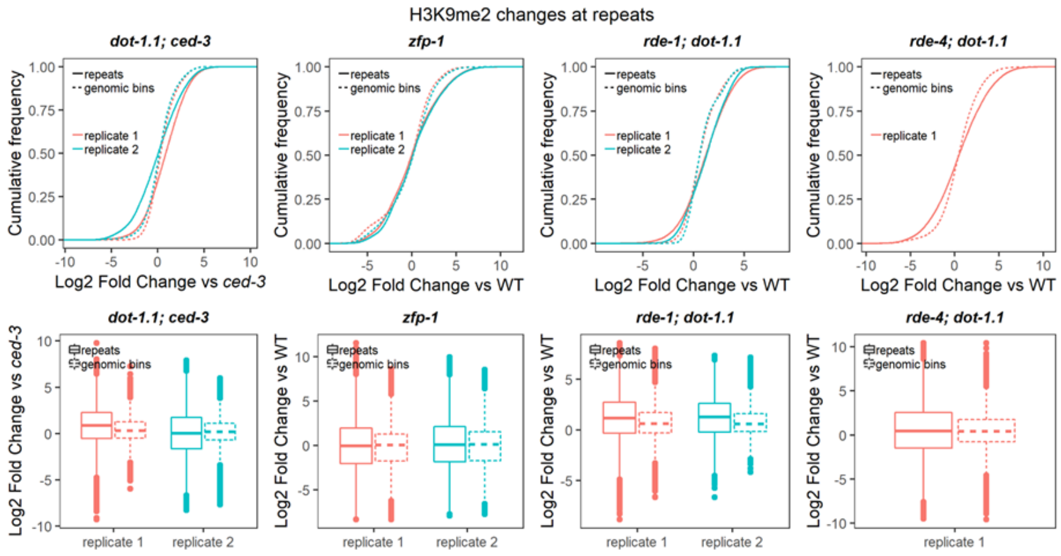

Figure S7. Deletion of DOT-1.1 leads to global increase in H3K9me2 at repeats. Shown are cumulative distribution plots and boxplots representing the fold change (log2) of H3K9me2 ChIP-seq RPKM value (input-normalized) at genomic repeats [6] between each mutant replicate and the average value in the background strain. Two-sample Kolmogorov-Smirnov (for cumulative distribution plots) and Wilcoxon rank sum (for boxplots) tests were performed to compare the cumulative changes between genomic repeat regions and those in non-overlapping genomic bins spanning the genome. Statistical significance (p-values < 2.2×10^{-16}) was found for all *dot-1.1; ced-3* and *rde-1; dot-1.1* mutant replicates analyzed.

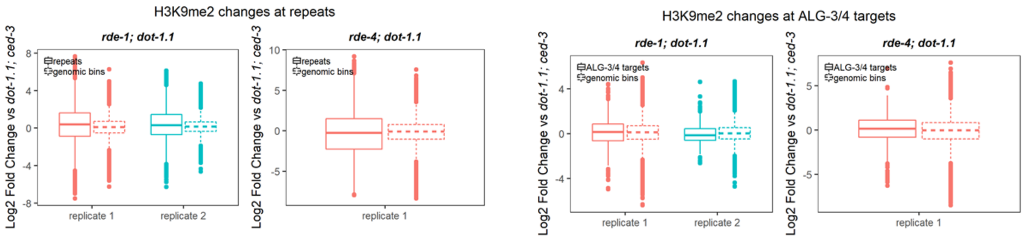

(a) (b)

Figure S8. Boxplots showing cumulative changes in the fold change (log2) of H3K9me2 ChIP-seq RPKM value (input-normalized) at repeats [6] (**a**) and ALG-3/4 targets [7] (**b**) between the *rde-1; dot-1.1* and *rde-4; dot-1.1* mutants and the average value in the *dot-1.1; ced-3* mutant. Wilcoxon rank sum tests were performed to compare the changes between either repeats or ALG-3/4 targets and those in non-overlapping genomic bins spanning the genome. Statistical significance (p-values ranging between < 2.2×10^{-16} and 3.47×10^{-12}) was found for all *rde-1; dot-1.1* and *rde-4; dot-1.1* mutant replicates analyzed.

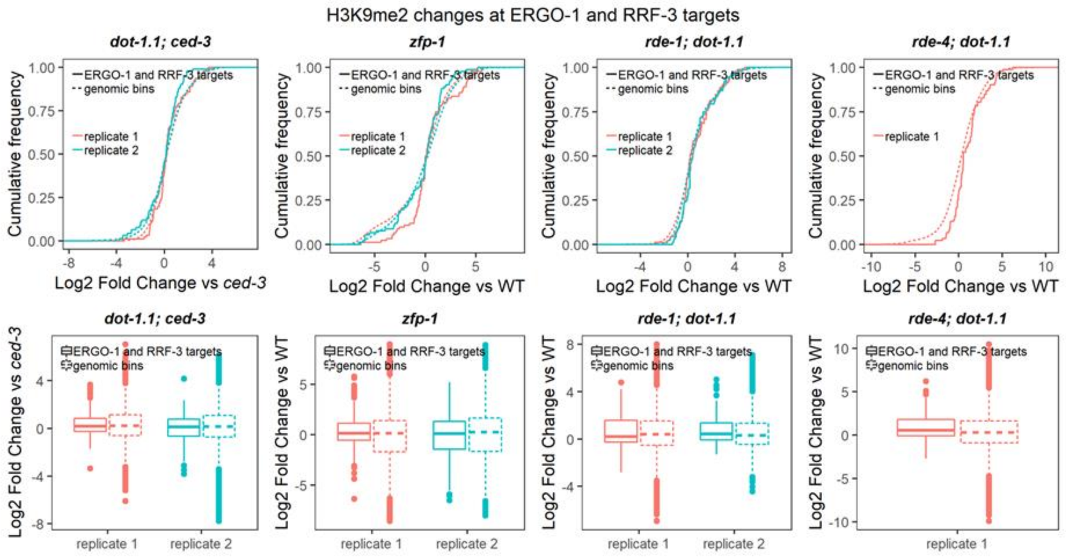

Figure S9. Deletion of DOT-1.1 does not significantly disrupt global H3K9me2 at ERGO-1 and RRF-3 targets. Shown are cumulative distribution plots and boxplots representing the fold change (log2) of H3K9me2 ChIP-seq RPKM value (input-normalized) at ERGO-1 and RRF-3 targets [8] between each mutant replicate and the average value in the background strain. Two-sample Kolmogorov-Smirnov (for cumulative distribution plots) and Wilcoxon rank sum (for boxplots) tests were performed to compare the cumulative changes between ERGO-1 and RRF-3 targets and those in non-overlapping genomic bins spanning the genome. Statistical significance (p-values < 0.002309) was observed only for the *rde-4; dot-1.1* mutant.

(a)

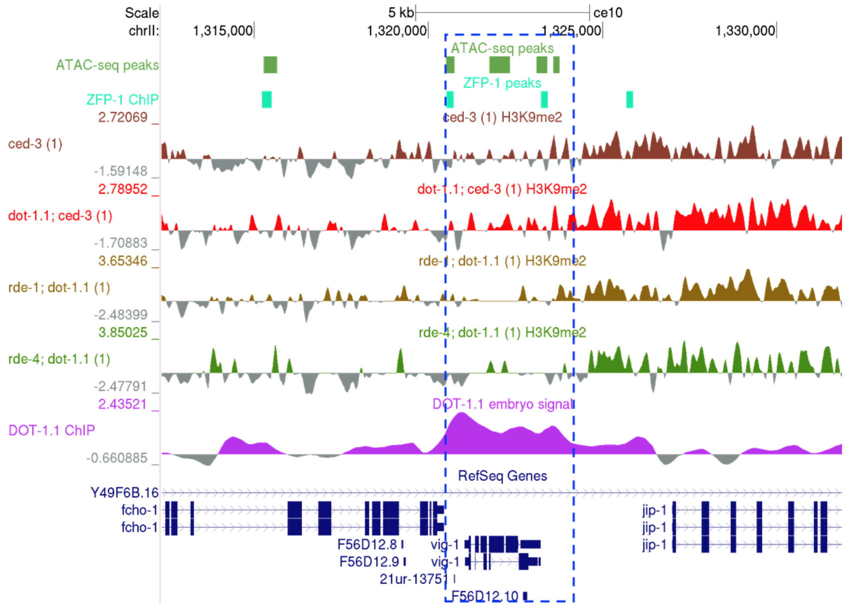

(b)

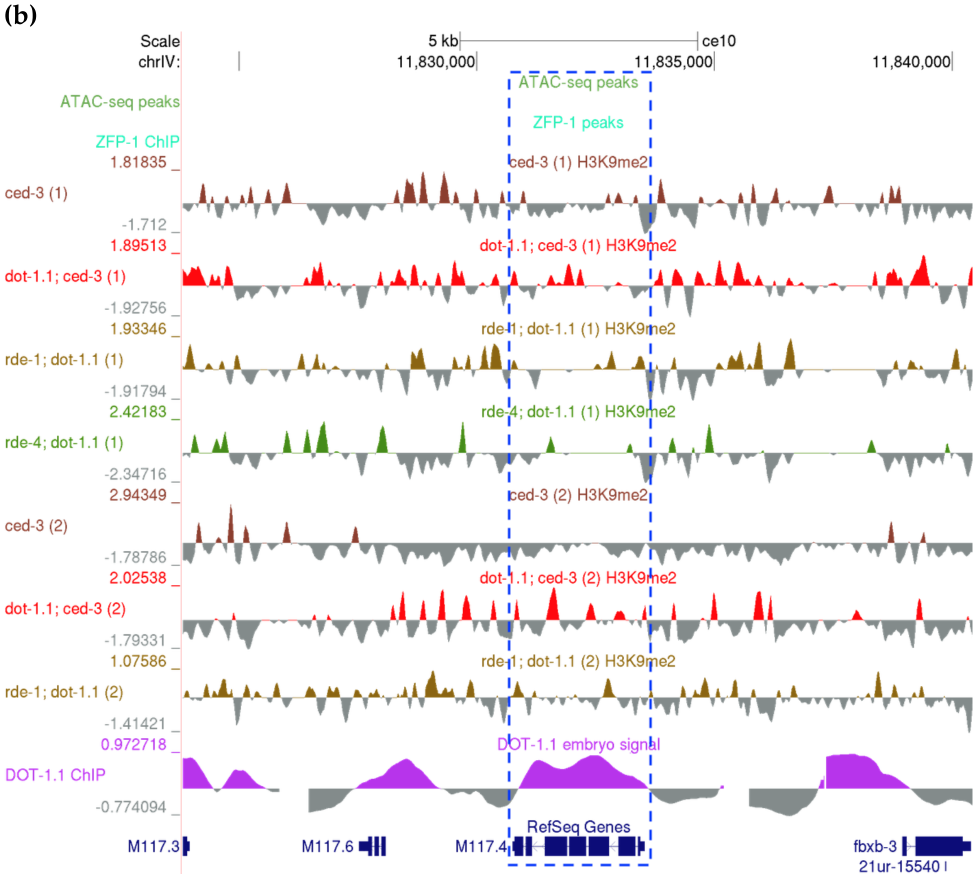

Figure S10. Examples of ALG-3/4 target genes that show RDE-1/4-dependent gain of H3K9me2 in the absence of DOT-1.1 (UCSC genome browser screenshots) (**a**) The *vig-1* gene contains intragenic enhancers. (**b**) The M117.4 gene is bound by DOT-1.1 in the embryo. ATAC-seq peaks: [4]. ZFP-1 peaks: modENCODE data, GEO submission GSE50301. H3K9me2 coverage tracks: our data. DOT-1.1 ChIP-chip signal: modENCODE data, GEO submission GSE37488.

References

1. Esse, R.; Gushchanskaia, E.S.; Lord, A.; Grishok, A. DOT1L complex suppresses transcription from enhancer elements and ectopic RNAi in Caenorhabditis elegans. *RNA* **2019**, *25*, 1259–1273.
2. Cecere, G.; Hoersch, S.; Jensen, M.B.; Dixit, S.; Grishok, A. The ZFP-1(AF10)/DOT-1 complex opposes H2B ubiquitination to reduce Pol II transcription. *Mol. Cell* **2013**, *50*, 894–907.
3. Rockman, M.V.; Kruglyak, L. Recombinational landscape and population genomics of Caenorhabditis elegans. *Plos Genet.* **2009**, *5*, e1000419.
4. Daugherty, A.C.; Yeo, R.W.; Buenrostro, J.D.; Greenleaf, W.J.; Kundaje, A.; Brunet, A. (2017). Chromatin accessibility dynamics reveal novel functional enhancers in C. elegans. *Genome Res.* **2017**, *27*, 2096–2107.
5. Evans, K.J.; Huang, N.; Stempor, P.; Chesney, M.A.; Down, T.A.; Ahringer, J. Stable Caenorhabditis elegans chromatin domains separate broadly expressed and developmentally regulated genes. *Proc. Natl Acad Sci Usa* **2016**, *113*, E7020–E7029.
6. McMurchy, A.N.; Stempor, P.; Gaarenstroom, T.; Wysolmerski, B.; Dong, Y.; Aussianikava, D.; Appert, A.; Huang, N.; Kolasinska-Zwierz, P.; Sapetschnig, A.; et al. A team of heterochromatin factors collaborates with small RNA pathways to combat repetitive elements and germline stress. *eLife* **2017**, *6*, e21666.
7. Conine, C.C., Batista, P.J., Gu, W., Claycomb, J.M., Chaves, D.A., Shirayama, M., and Mello, C.C. (2010). Argonautes ALG-3 and ALG-4 are required for spermatogenesis-specific 26G-RNAs and thermotolerant sperm in Caenorhabditis elegans. *Proc. Natl. Acad. Sci. USA* **2010**, *107*, 3588–3593.

8. Vasale, J.J.; Gu, W.; Thivierge, C.; Batista, P.J.; Claycomb, J.M.; Youngman, E.M.; Duchaine, T.F.; Mello, C.C.; Conte, D., Jr. Sequential rounds of RNA-dependent RNA transcription drive endogenous small-RNA biogenesis in the ERGO-1/Argonaute pathway. *Proc. Natl. Acad. Sci. USA* **2010**, *107*, 3582–3587.

© 2020 by the authors. Licensee MDPI, Basel, Switzerland. This article is an open access article distributed under the terms and conditions of the Creative Commons Attribution (CC BY) license (http://creativecommons.org/licenses/by/4.0/).
